# Supplementary material for: Identification of fidelity-governing factors in human recombinases DMC1 and RAD51 from cryo-EM structures
Source: Nat Commun. 2021 Jan 14;12:115. doi: 10.1038/s41467-020-20258-1 (PMC7809202; doi:10.1038/s41467-020-20258-1)
Supplement: Supplementary file 1 — Supplementary Information [file 41467_2020_20258_MOESM1_ESM.pdf]

Supplementary Information

Identification of fidelity-governing factors in human recombinases DMC1 and RAD51 from cryo-EM structures

Shih-Chi Luo[1,#], Hsin-Yi Yeh[2,#], Wei-Hsuan Lan[3], Yi-Min Wu[1], Cheng-Han Yang[1], Hao-Yen Chang[2], Guan-Chin Su[2], Chia-Yi Lee[2], Wen-Jin Wu[1], Hung-Wen Li[3], Meng-Chiao Ho[1,2,*], Peter Chi[1,2,*], and Ming-Daw Tsai[1,2,*]

[1]Institute of Biological Chemistry, Academia Sinica, Taipei, Taiwan

[2]Institute of Biochemical Sciences, National Taiwan University, Taipei, Taiwan

[3]Department of Chemistry, National Taiwan University, Taipei, Taiwan

[#]These two authors contributed equally.

[*]e-mail: joeho@gate.sinica.edu.tw; peterhchi@ntu.edu.tw; mdtsai@gate.sinica.edu.tw

This file includes:

Supplementary Figures 1-8

Supplementary Tables 1-2

References for Supplementary Information

Supplementary Fig. 1 (3 pages). Flow chart of the cryo-EM structure determination strategy and resolution assessment of DMC1-ssDNA (Fig. 1-1), DMC1-dsDNA (Fig. 1-2), DMC1-dsDNA (mismatched, Fig. 1-3), DMC1-Q244M-dsDNA (Fig. 1-4), and RAD51-VpDg-dsDNA (Fig. 1-5).

a, The workflow for structural determination. A representative cryo-EM micrograph and a representative 2D class average are shown in the top panel. After 3D classification, sub-classes with higher resolution were selected for further refinement. **b**, The final average map resolution for the overall density of the filaments is shown as calculated in RELION by the gold standard technique (FSC = 0.143). FSC curves of the refined model versus summed map (black), refined model versus half map 1 (red), and refined model versus half map 2 (green) were calculated by Phenix. **c**, Helical reconstruction density maps. The model of the ssDNA is shown in red, while the dsDNA is shown in red (invading strand) and blue (complementary strand).

Supplementary Fig. 1-1:

DMC1-ssDNA flow chart of structural determination procedures

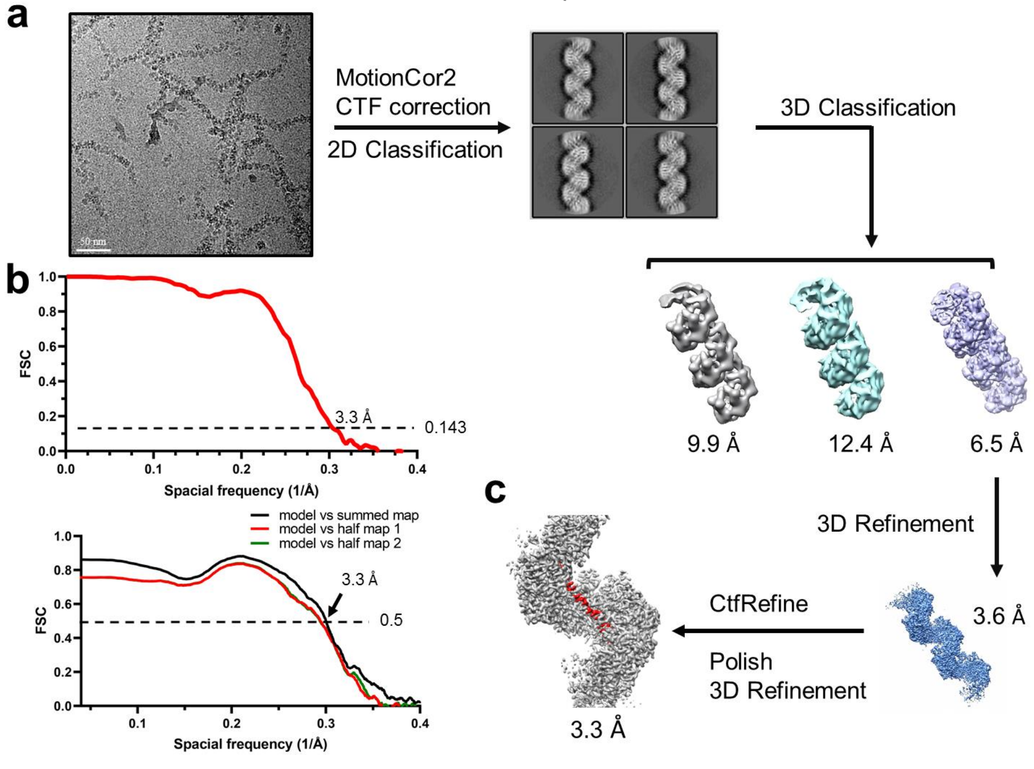

Supplementary Fig. 1-2:
DMC1-dsDNA flow chart of structural determination procedures

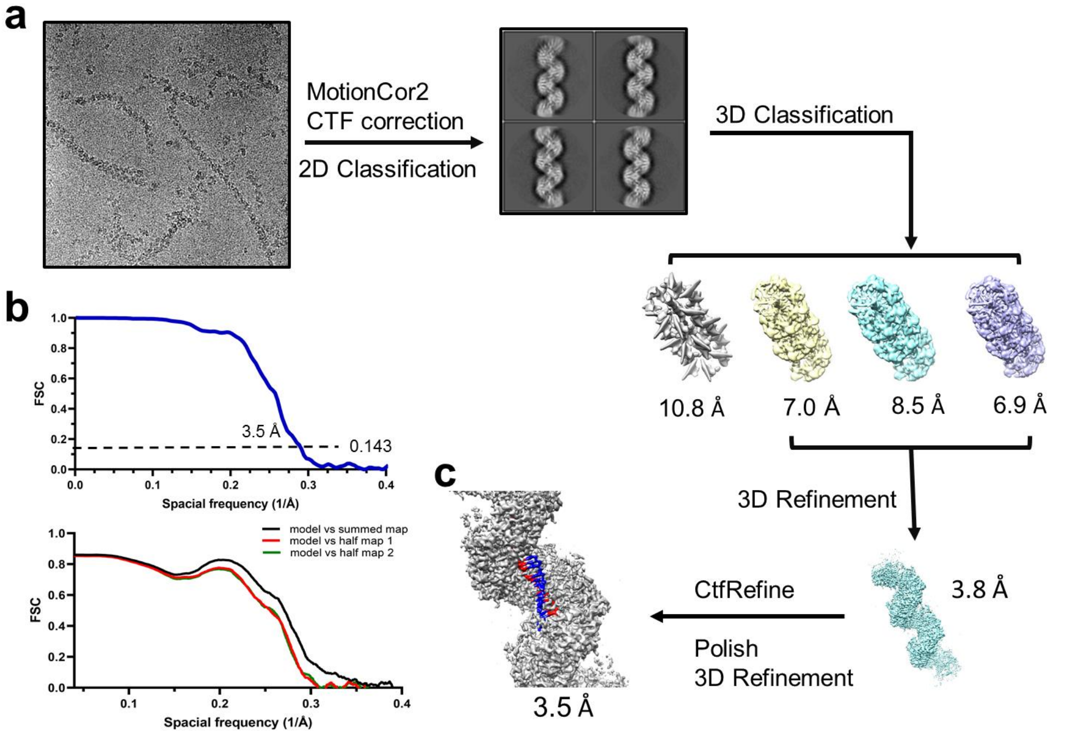

Supplementary Fig. 1-3:
DMC1-dsDNA (mismatched) flow chart of structural determination procedures

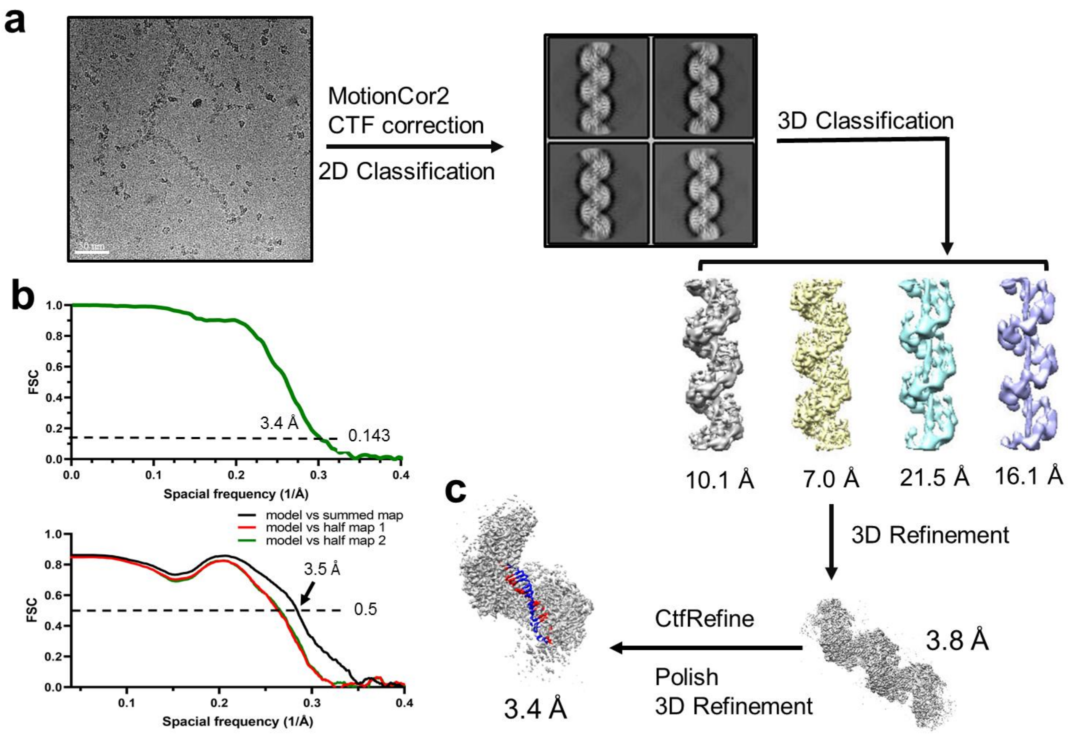

Supplementary Fig. 1-4:
DMC1Q244M-dsDNA flow chart of structural determination procedures

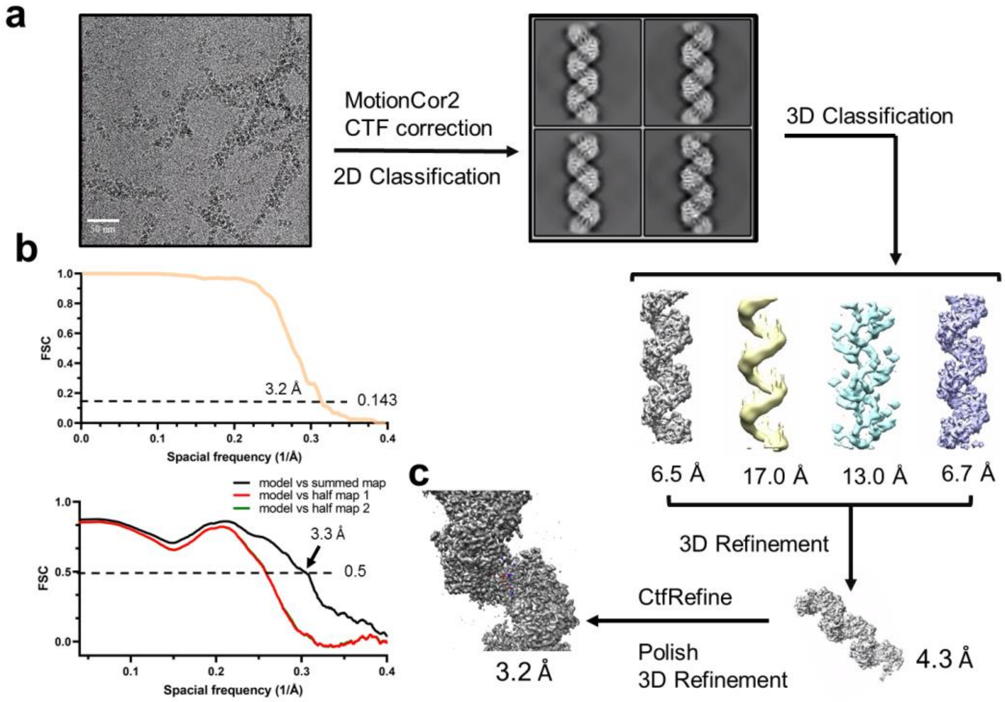

Supplementary Fig. 1-5:
RAD51VpDg-dsDNA flow chart of structural determination procedures

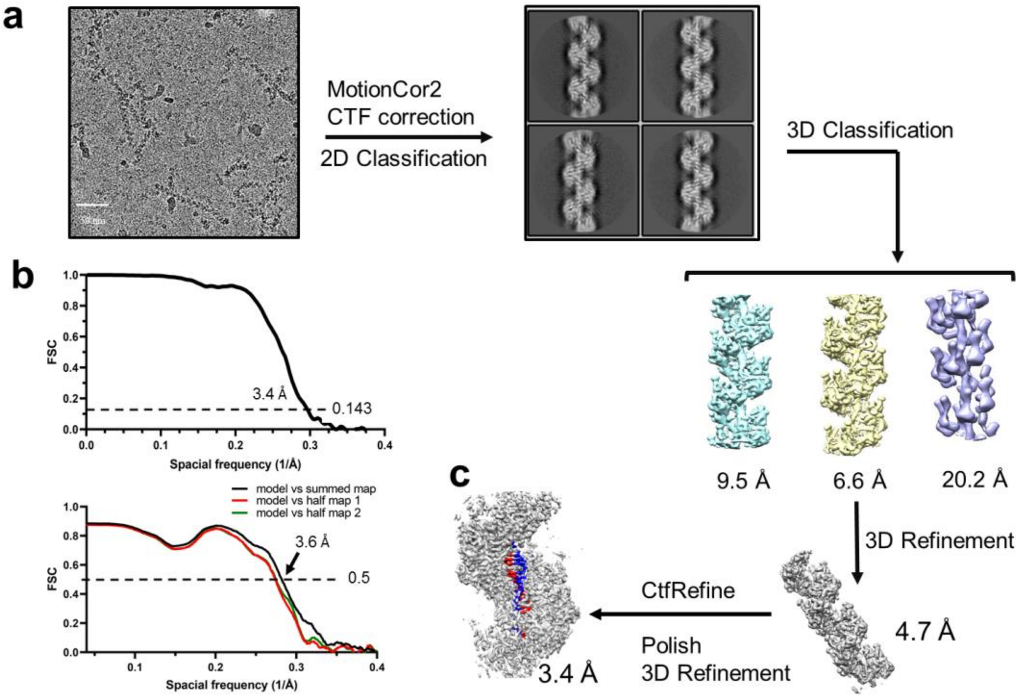

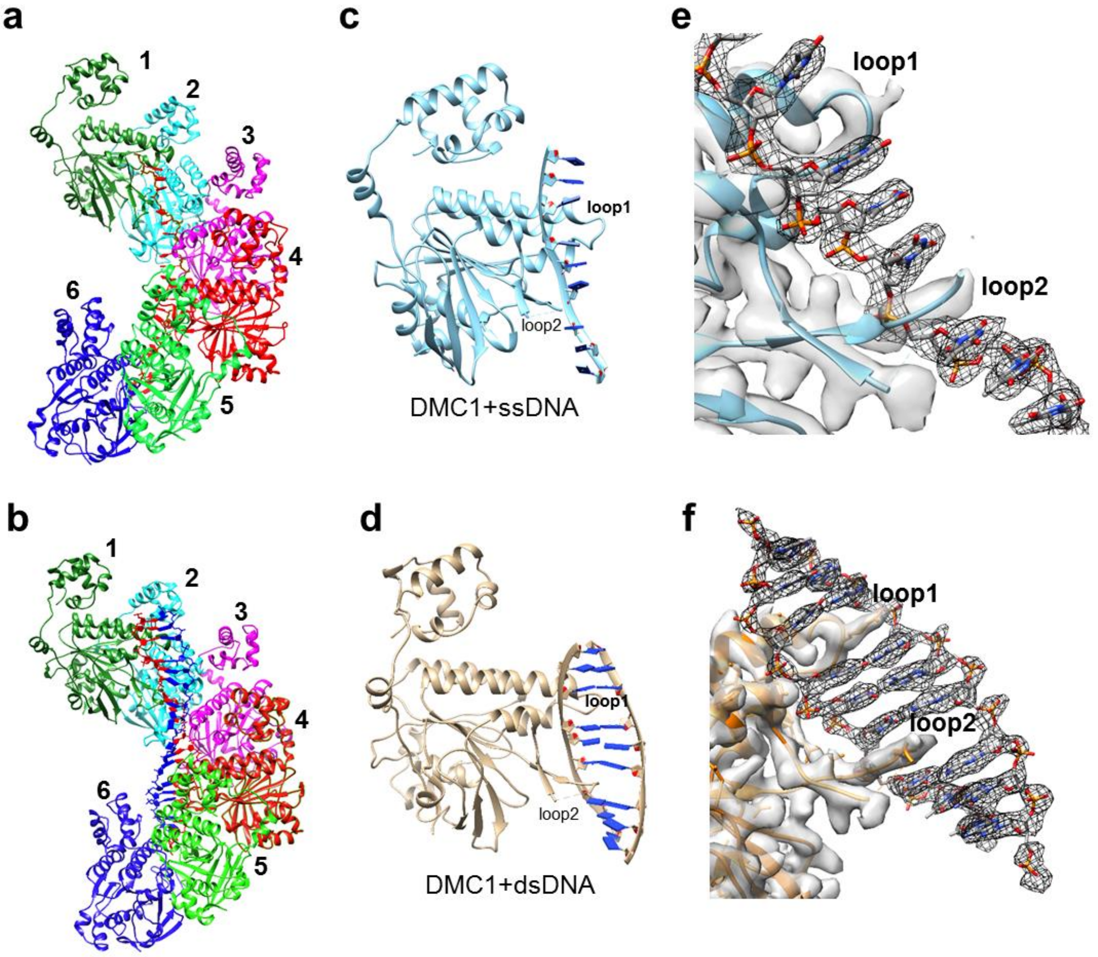

Supplementary Fig. 2. Structures of the DMC1 presynaptic and postsynaptic filaments.

a-b, Atomic models of DMC1 presynaptic (a) and postsynaptic (b) filament containing the 6-protomer units with ssDNA/dsDNA. The invading strand ssDNA and the complementary strand from the homologous dsDNA are red and blue, respectively. Each DMC1 protomer is colored differently. **c-d**, Structures of single protomers from a and b, respectively, including part of the bound DNA. The backbone RMSD between presynaptic and postsynaptic DMC1 promotor is 0.92 Å out of a total of 308 C-alpha atoms. **e-f**, Cryo-EM maps of the DNA region superimposed with the atomic models of DNA from c and d, respectively.

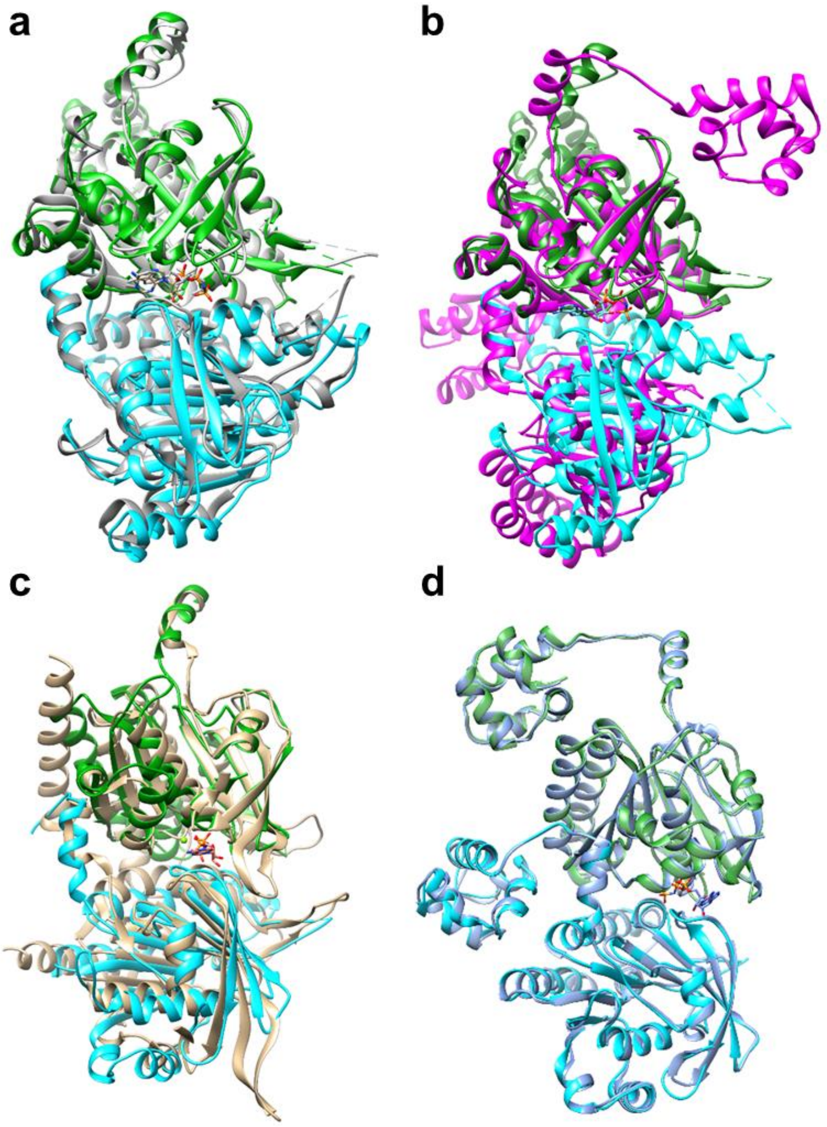

Supplementary Fig. 3. Structural comparison of protomer interface between DMC1 and other homologous recombinases.

a, Superposition of human DMC1 (in green and cyan colors) and human RAD51 (all in gray, PDB code 5H1B[1]) presynaptic filaments. **b**, Superposition of human DMC1 and *Saccharolobus solfataricus* RadA (all in magenta, PDB code 2ZUB[2]) presynaptic filaments. **c**, Superposition of human DMC1 and *E. coli* RecA (wheat, PDB code 3CMW[3]) presynaptic filaments. **d**, Superposition of DMC1 protomers from presynaptic and postsynaptic complexes (this study). The two promoters of presynaptic DMC1 are colored in green and cyan, while the postsynaptic DMC1 complex is colored in cornflower blue. The AMP-PNP molecule is shown as a stick model. Like the other recombinases, the molecular architecture of DMC1 presynaptic filaments shows a highly conserved ATP binding site at the interface between two protomers. The same architecture is retained in the DMC1 postsynaptic complex (cornflower blue) (d).

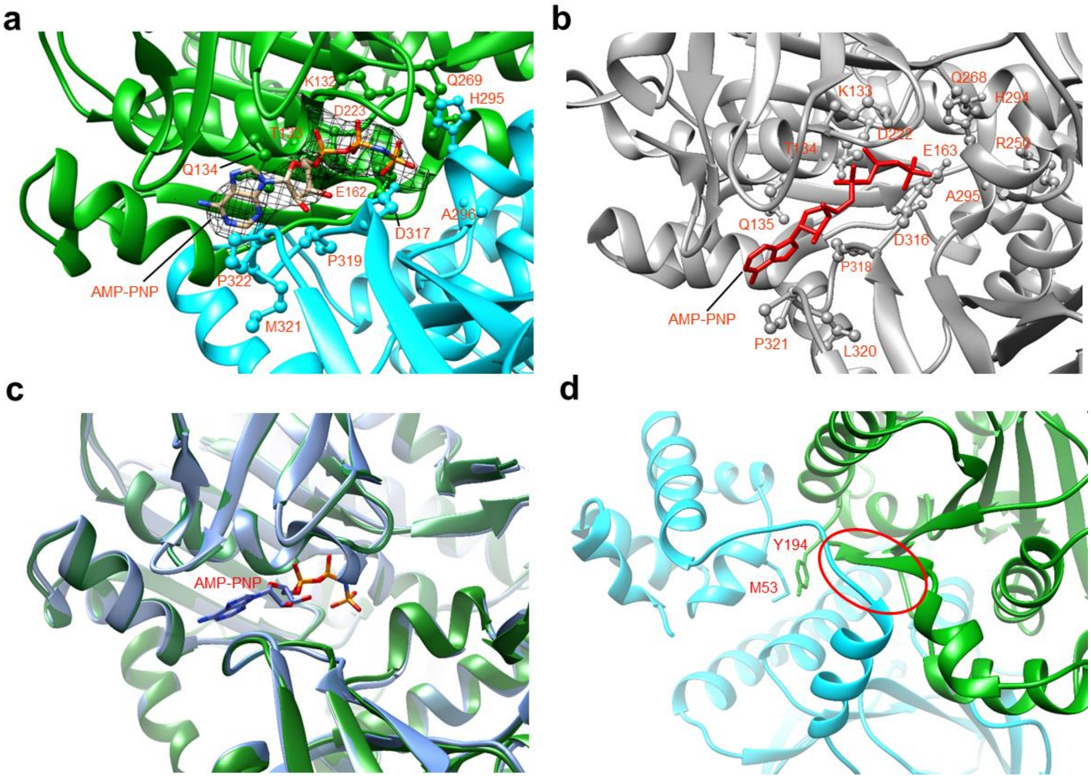

Supplementary Fig. 4. Structural analysis of the DMC1 ATP binding site.
a, ATP analog, AMP-PNP binds at the interface of two neighboring protomers (green and cyan) of the DMC1 presynaptic complex. The EM density map of AMP-PNP (stick model) is shown as black mesh. Key residues involved in nucleotide binding are labeled and shown in ball and stick. AMP-PNP is clearly visible in the EM density map and is sandwiched between the α/β ATPase cores of two adjacent DMC1 protomers in a buried environment. The Walker A motif (residues G126 to T133) and Walker B motif (residues L219 to D223) from one protomer wraps around the triphosphates. The other part of the sandwich involves six residues (P322, M321, P319, D317, A296, and H295) from the neighboring protomer of DMC1. **b**, Key residues involved in the nucleotide binding cores of RAD51 (grey, PDB code 5H1B[1]) are labeled and shown in ball and stick. These interactions are similar to those in the corresponding DMC1 complex. **c**, Comparison of the DMC1 presynaptic (green) and postsynaptic (cornflower blue) nucleotide binding pockets. For clarity, only AMP-PNP from the presynaptic complex is shown. Superposition between the DMC1 presynaptic and postsynaptic complexes also demonstrate structure similarity. **d**, The other two conserved interfaces in DMC1 eukaryotic orthologues. One is the hydrophobic packing between Y194 and M53, consistent with the previous report that DMC1 Y194 is a potential candidate interacting with N-terminal domain from the neighboring protomer[4]. Another one (red circle) is composed of the linker region between the N-terminal domain and the ATPase domain packing against the central β-sheet of the ATPase domain in the neighboring protomer. The structures shown are from the presynaptic complex but are similar for the postsynaptic complex.

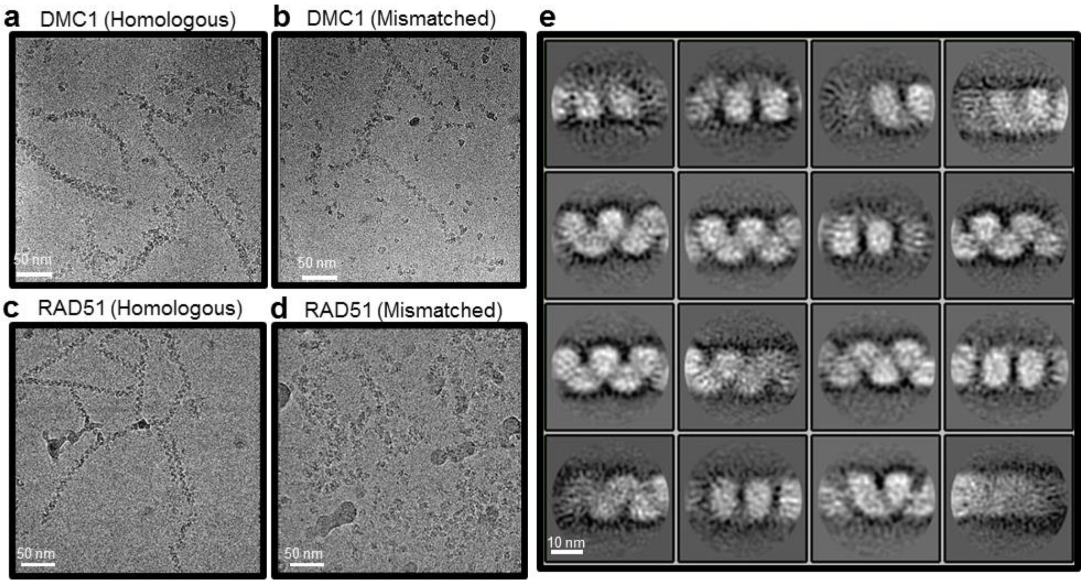

Supplementary Fig. 5. Raw images of matched and mismatched filaments.
a, DMC1 with homologous dsDNA. **b**, DMC1 with mismatched dsDNA. **c**, RAD51 with homologous dsDNA. The 2D classes from a-c are shown in Supplementary Figure 1. **d,e**, RAD51 with mismatched dsDNA shows irregular filaments and irregular 2D class averages, respectively. The micrographs shown in a-d are representative of 5057, 3191, 1353, and 538, respectively. The 2D class averages shown in e are from one experimental dataset, and are reproducible in three repeated calculations.

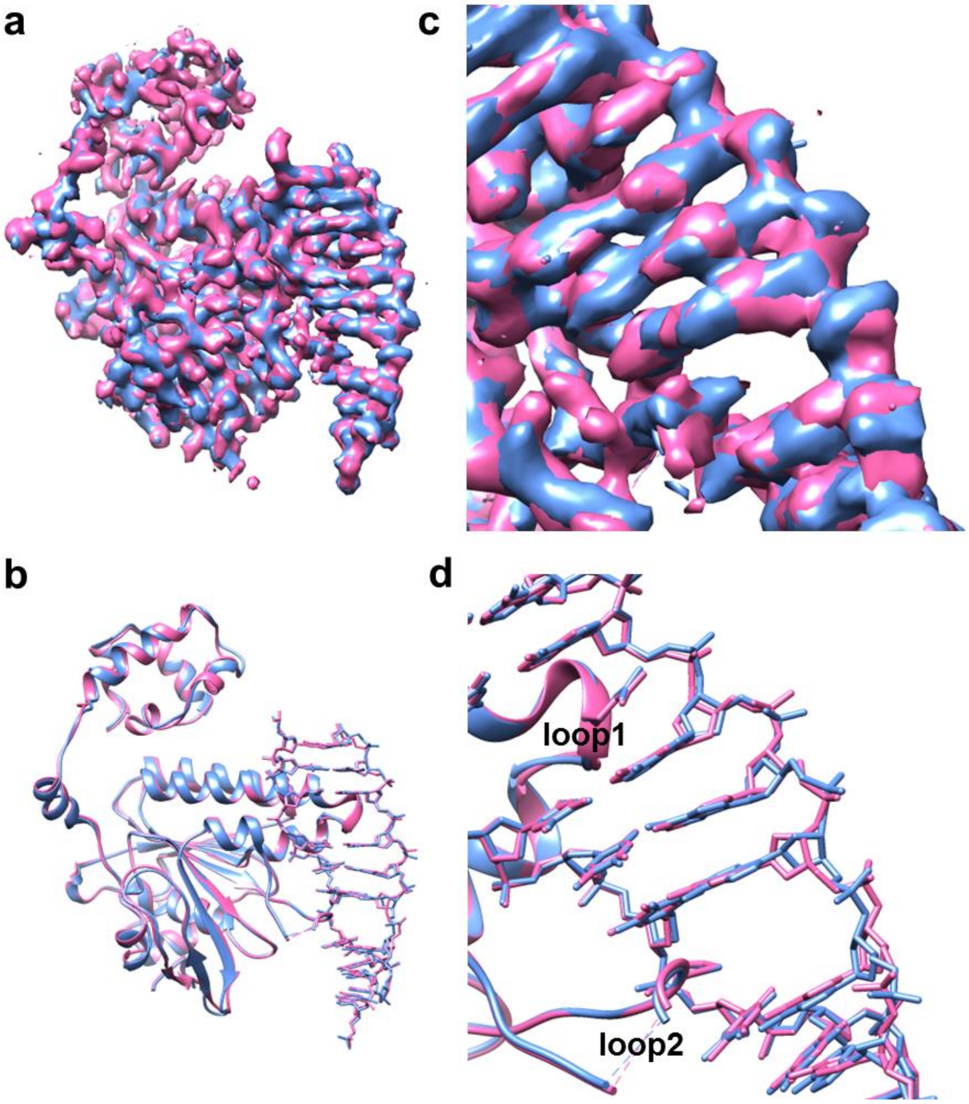

Supplementary Fig. 6. Similarity between the structural properties of homologous and mismatched DMC1-dsDNA filaments.

Superposition of the homologous (cornflower blue) and mismatched (hot pink) DMC1-dsDNA filaments (single protomer) on 3D density maps (**a, c**) or atomic models (**b, d**). The Loop 1, Loop 2 and part of the bound DNA are highlighted. The backbone RMSD between homologous and mismatched DMC1 protomers is 0.53 Å out of a total of 309 C-alpha atoms.

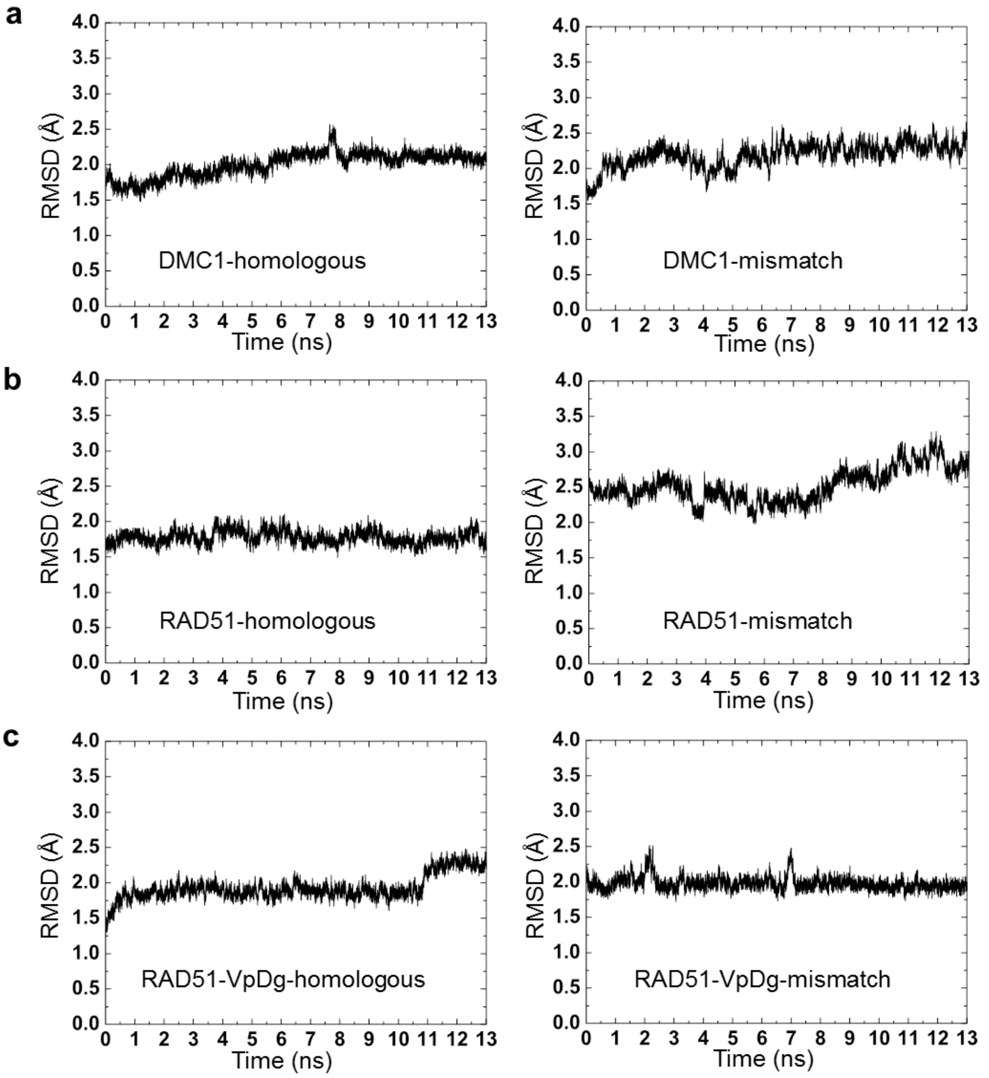

Supplementary Fig. 7. The DNA RMSD of homologous and mismatched filaments obtained from final 13 ns MD simulations.

For each simulation, the DNA molecules were aligned and the RMSD values (in Å), relative to the starting structural model of 9 base-pairs, 574 atoms including hydrogen, were plotted over time for (**a**) DMC1, (**b**) RAD51, and (**c**) RAD51-VpDg.

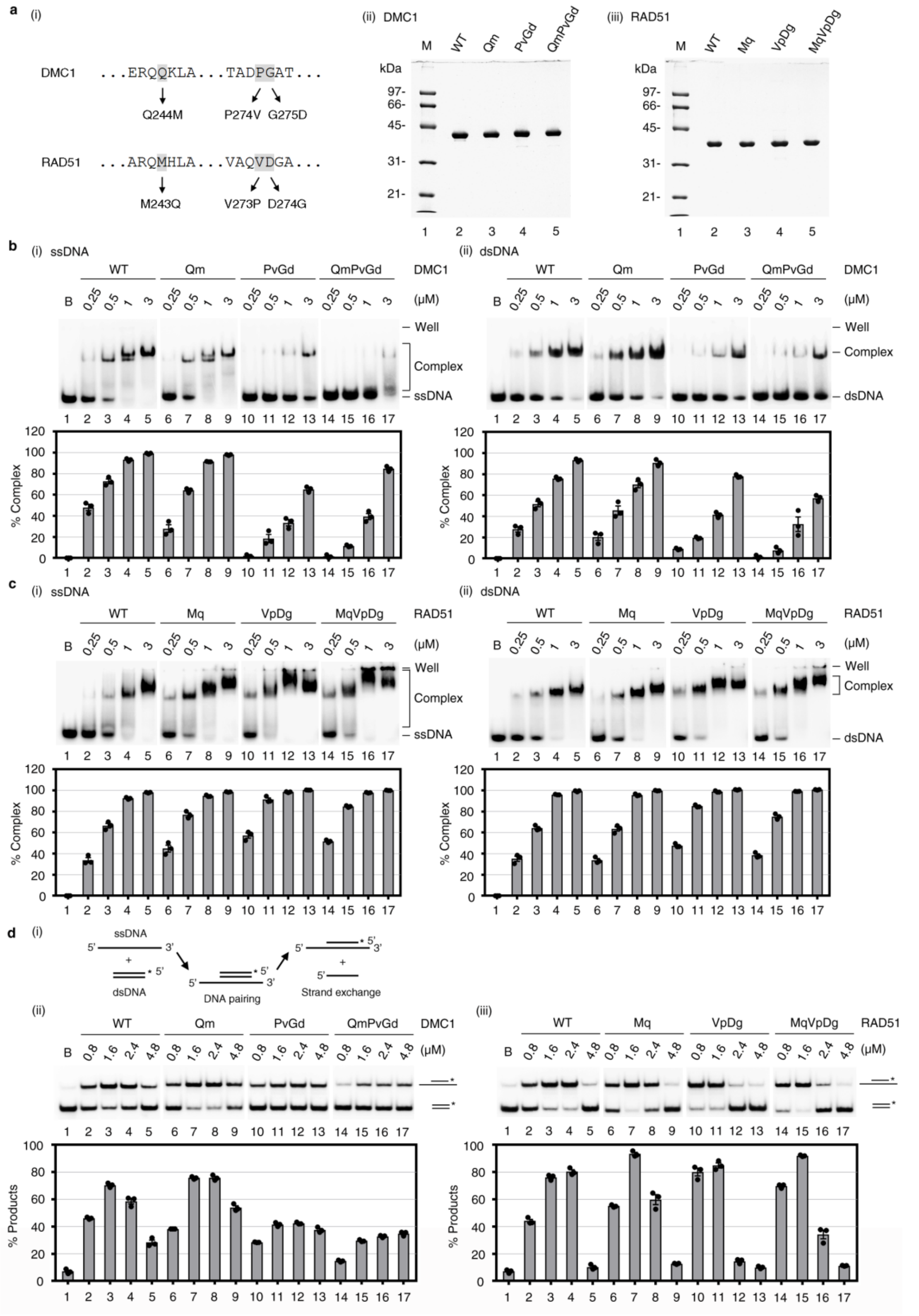

Supplementary Fig. 8. Biochemical characteristics of DMC1 and RAD51 variants for Loop 1 and Loop 2 residues. a, Purification of the mutant variants. (i) DMC1 and RAD51 mutations generated in this study. Purified DMC1 (ii) and RAD51 (iii) mutant variants (2 μg) was analyzed on a 12% SDS-PAGE and were reproducible in two repeated experiments. **b**, **c**, DNA-binding activity of DMC1 (**b**) and RAD51 (**c**) variants. Panels (i) and (ii) represent the ssDNA- and dsDNA-binding activities, respectively.

The results were graphed, and the % complex was calculated by the decreased percentage of free DNA. **d**, DNA strand exchange assay. (i) The reaction schematics. (ii) and (iii) show the DNA strand exchange activity of DMC1 and RAD51 variants, respectively. The ^{32}P label is denoted by an asterisk. The results were graphed. **b-d,** The error bars represent the standard error of mean (±s.e.m.) based on three independent experiments (n=3). Overall, all mutants exhibit the same purity (>95%) as the wild-type, and comparable ssDNA and dsDNA binding activities and strand exchange activities, except that DMC1 PvGd and QmPvGd lost approximately 40% and 60%, respectively, of both DNA binding and strand exchange activities. Source data are provided in the Source Data file. Due to the substantial perturbation of its functions, QmPvGd was omitted from the mismatch tolerance experiments.

Supplementary Table 1. Cryo-EM data collection, processing, refinement and validation statistics

	#1 DMC1-ssDNA	#2 DMC1-dsDNA	#3 DMC1-dsDNA (mismatch)	#4 DMC1-Q244M-dsDNA	#5 RAD51-VpDg-dsDNA
**Accession code**					
EMDB	30311	30308	30309	30366	30310
PDB	7C9C	7C98	7C99	7CGY	7C9A
**Data collection and processing**					
Magnification	165,000	165,000	165,000	165,000	165,000
Voltage (kV)	300	300	300	300	300
Electron exposure (e–/Å^2)	50	50	50	50	50
Defocus range (μm)	-1.5 to-2.5	-1.5 to -2.5	-1.5 to-2.5	-1.5 to -2.5	-1.5 to-2.5
Pixel size (Å)	0.84	0.84	0.84	0.84	0.84
Symmetry imposed	Helical	Helical	Helical	Helical	Helical
Total particle images (no.)	192,466	223,902	140,823	643,100	136,657
Box size (pixel)	384	384	384	384	384
Rise (Å)	15.71	15.72	15.72	16.00	15.80
Twist	55.48°	55.59°	55.49°	55.93°	56.18°
Map resolution (Å)	3.33	3.47	3.36	3.20	3.43
FSC threshold	0.143	0.143	0.143	0.143	0.143
Map resolution range (Å)	3.3-8.0	3.5-8.0	3.4-7.4	3.2-8.0	3.4-6.6
**Refinement**					
Initial model used (PDB)	5H1B	5H1C	7C98	7C98	5H1C
Model resolution	3.33	3.69	3.52	3.3	3.55
(Masked, Å) FSC threshold	0.5	0.5	0.5	0.5	0.5
Map sharpening *B* factor (Å^2)	-73	-75	-71	-98	-80
Model composition					
Non-hydrogen atoms	7,455	7,695	7,659	7,215	7,593
Protein residues	924	927	927	915	933
Nucleotide	9	18	18	0	18
Lig.: AMP-PNP, Ca	3, 3	3, 3	3, 3	3, 3	3, 3
B factors (Å^2)					
Protein	54.47	93.02	69.32	68.12	68.7
Nucleic acid	45.52	68.40	56.72	-	69.58
Ligand	35.63	69.45	50.34	53.52	51.58
R.m.s. deviations					
Bond lengths (Å)	0.007	0.013	0.006	0.006	0.005
Bond angles (°)	0.880	0.906	0.878	1.013	0.882
Validation					
MolProbity score	1.68	1.95	1.92	1.82	1.8
Clash score	5.78	9.01	9.93	8.62	8.52
Poor rotamers (%)	0.40	0	0	0.13	0
Ramachandran plot					
Favored (%)	94.74	92.57	93.99	94.91	95.11
Allowed (%)	5.26	7.43	6.01	4.87	0
Disallowed (%)	0	0	0	0.22	0

Supplementary Table 2. DNA oligonucleotide used in this study

Oligo No.	Sequence
Oligo1<br>dsDNA (-)	5'TTATGTTCATTTTTTATATCCTTTACTTTATTTTCTCTGTTTATTCATTTAC<br>TTATTTTGTATTATCCTTATCTTATTTA-3'
Oligo2<br>dsDNA (+)	5'TAAATAAGATAAGGATAATACAAAATAAGTAAATGAATAAACAGAGAA<br>AATAAAGTAAAGGATATAAAAAATGAACATAA-3'
Oligo3<br>dsDNA (-)	5'ACGGGCAACAGCTGA-3'
Oligo4<br>dsDNA (+)	5'TCAGCTGTTGCCCGT-3'
Oligo5<br>dsDNA (+)	5'TCAGCTGT***C***GCCCGT-3'
Oligo6<br>dsDNA (-)	5'CTTTATTTTCTCTGTTTATTCATTTACTTATTTTGTATTA-3'
Oligo7<br>dsDNA (+)	5'TAATACAAAATAAGTAAATGAATAAACAGAGAAAATAAAG-3'
Oligo8<br>ssDNA (+)	5'<u>AAATGAACATAAAGTAAATAAGTATAAGGATAATACAAAA</u>TAAGTAAA<br>TGAATAAACATAGAAAATAAAGTAAAGGATATAAA-3'
Oligo9<br>ssDNA (+)	5'<u>AAATGAACATAAAGTAAATAAGTATAAGGATAAT***G***CAAAA</u>TAAGTAAA<br>TGAATAAACATAGAAAATAAAGTAAAGGATATAAA-3'
Oligo10<br>dsDNA (-)	5'TTTTGTATTATCCTTATACTTATTTACTTTATGTTCATTT-3'-Cy5
Oligo11<br>dsDNA (+)	Cy3-5'AAATGAACATAAAGTAAATAAGTATAAGGATAATACAAAA-3'
Oligo12<br>hybrid DNA (-)	Cy5-5'-GCCTCGCTGCCGTCGCCA-3'-bio
Oligo13<br>hybrid DNA (+)	5'TGGCGACGGCAGCGAGGCTTAATTCATCTTCATTATCTCAGTTCTATCA<br>ATCTTAATCTAATACTC<u>TTACCTTCCCTCCCT</u>CTGTTTACTATATTCTTA-3'
Oligo14<br>hybrid DNA (+)	5'TGGCGACGGCAGCGAGGCTTAATTCATCTTCATTATCTCAGTTCTATCA<br>ATCTTAATCTAATACTC<u>TTACCTTCCC***A***CCCT</u>CTGTTTACTATATTCTTA-3'
Oligo15<br>hybrid DNA (+)	5'TGGCGACGGCAGCGAGGCTTAATTCATCTTCATTATCTCAGTTCTATCA<br>ATCTTAATCTAATACTC<u>TTACCTTCCCTC</u>ATACTGTTTACTATATTCTTA-3'
Oligo16<br>dsDNA (-)	Cy3-5'-TTCAGGGAGGGAAGGTAATTTT TGTTTTTTATTTTTTTTT-3'
Oligo17<br>dsDNA (+)	5'AAAAAAAAATAAAAAACAAAAATTACCTTCCCTCCCTGAA-3'

Notes: (1). The bold and italic letters on the sequences of Oligos 5, 9, and 14 represent the mismatched bases corresponding to their dsDNA donors. (2). The sequences with underline are homologous with their dsDNA donor.

References for Supplementary Information

1. Xu J, Zhao L, Xu Y, Zhao W, Sung P, Wang HW. Cryo-EM structures of human RAD51 recombinase filaments during catalysis of DNA-strand exchange. *Nat Struct Mol Biol* **24**, 40-46 (2017).

2. Chang Y-W*, et al.* Three New Structures of Left-Handed RadA Helical Filaments: Structural Flexibility of N-Terminal Domain Is Critical for Recombinase Activity. *PLOS ONE* **4**, e4890 (2009).

3. Chen Z, Yang H, Pavletich NP. Mechanism of homologous recombination from the RecA–ssDNA/dsDNA structures. *Nature* **453**, 489-494 (2008).

4. Kinebuchi T, Kagawa W, Kurumizaka H, Yokoyama S. Role of the N-terminal domain of the human DMC1 protein in octamer formation and DNA binding. *J Biol Chem* **280**, 28382-28387 (2005).
