# Supplementary material for: Identification of fidelity-governing factors in human recombinases DMC1 and RAD51 from cryo-EM structures
Source: Nat Commun. 2021 Jan 14;12:115. doi: 10.1038/s41467-020-20258-1 (PMC7809202; doi:10.1038/s41467-020-20258-1)
Supplement: Supplementary file 2 — Reporting Summary [file 41467_2020_20258_MOESM2_ESM.pdf]

nature research

Corresponding author(s): Meng-Chiao Ho, Peter Chi, and Ming-Daw Tsai

Last updated by author(s): Nov 12, 2020

Reporting Summary

Nature Research wishes to improve the reproducibility of the work that we publish. This form provides structure for consistency and transparency in reporting. For further information on Nature Research policies, see our Editorial Policies and the Editorial Policy Checklist.

Statistics

For all statistical analyses, confirm that the following items are present in the figure legend, table legend, main text, or Methods section.

n/a	Confirmed	
☐	☒	The exact sample size (*n*) for each experimental group/condition, given as a discrete number and unit of measurement
☐	☒	A statement on whether measurements were taken from distinct samples or whether the same sample was measured repeatedly
☐	☒	The statistical test(s) used AND whether they are one- or two-sided<br>*Only common tests should be described solely by name; describe more complex techniques in the Methods section.*
☐	☒	A description of all covariates tested
☐	☒	A description of any assumptions or corrections, such as tests of normality and adjustment for multiple comparisons
☐	☒	A full description of the statistical parameters including central tendency (e.g. means) or other basic estimates (e.g. regression coefficient) AND variation (e.g. standard deviation) or associated estimates of uncertainty (e.g. confidence intervals)
☐	☒	For null hypothesis testing, the test statistic (e.g. *F*, *t*, *r*) with confidence intervals, effect sizes, degrees of freedom and *P* value noted<br>*Give P values as exact values whenever suitable.*
☒	☐	For Bayesian analysis, information on the choice of priors and Markov chain Monte Carlo settings
☒	☐	For hierarchical and complex designs, identification of the appropriate level for tests and full reporting of outcomes
☒	☐	Estimates of effect sizes (e.g. Cohen's *d*, Pearson's *r*), indicating how they were calculated

Our web collection on statistics for biologists contains articles on many of the points above.

Software and code

Policy information about availability of computer code

Data collection	Software used for the particular experiments:<br>Phosphorimaging analysis (Bio-Rad Personal Molecular Imager): EMSA and DNA strand exchange<br>Gene5 2.07 spectrofluorometer (BioTek SynergyHTX multi-mode reader): fluorescence-based DNA strand exchange<br>LABVIEW 8.6: Single molecule triplex state stability experiments image acquisition<br>Cryo-EM data were acquired on a Titan Krios G3 (Thermo Fisher) microscope operated at 300 keV, equipped with a GIF Quantum K2 dector system (Gatan). Automated data acquisition was carried out using EPU software (Thermo Fisher).
Data analysis	Quantitative analyses for phosphorimages were performed using Bio-Rad Quantity One 4.6.9.<br>One-way ANOVA and unpaired t-test were performed using GraphPad Prism 7 (GraphPad Software).<br>Repeated measure ANOVA was performed using Statistical Product and Service Solutions.<br>Single-molecule data analysis for automatic spot detection and co-localization was performed using MATLAB.<br>Single-molecule data analysis for fitting was performed using Origin.<br>CryoEM data processing was motion-corrected and dose-weighted using RELION3.0's own implementation. CryoEM structure refinement was done with Rosetta. The refined atomic models were further validated and manually inspected using PHENIX and COOT.

For manuscripts utilizing custom algorithms or software that are central to the research but not yet described in published literature, software must be made available to editors and reviewers. We strongly encourage code deposition in a community repository (e.g. GitHub). See the Nature Research guidelines for submitting code & software for further information.

Data

Policy information about availability of data

All manuscripts must include a data availability statement. This statement should provide the following information, where applicable:

- Accession codes, unique identifiers, or web links for publicly available datasets
- A list of figures that have associated raw data
- A description of any restrictions on data availability

The refined coordinates and corresponding cryo-EM maps have been deposited in the Protein Data Bank and the Electron Microscopy Data Bank under accession codes PDB-7C9C [https://doi.org/10.2210/pdb7C9C/pdb] and EMD-30311 (DMC1 pre-synaptic complex), PDB-7C98 [https://doi.org/10.2210/pdb7C98/pdb] and EMD-30308 (DMC1 post-synaptic complex), PDB-7C99 [https://doi.org/10.2210/pdb7C99/pdb] and EMD-30309 (DMC1 post-synaptic complex with mismatch), PDB-7CGY [https://doi.org/10.2210/pdb7CGY/pdb] and EMD-30366 (DMC1-Q244M mutant post-synaptic complex), PDB-7C9A [https://doi.org/10.2210/pdb7C9A/pdb] and EMD-30310 (RAD51-V273P, D274G mutant post-synaptic complex). All other relevant data are described in the Supplementary Information or the Source Data file provided with this paper. The Source Data file consists of raw data and uncropped gel images for Figure 4 and Supplementary Figure 8. Any additional data related to this paper are available upon request.

Field-specific reporting

Please select the one below that is the best fit for your research. If you are not sure, read the appropriate sections before making your selection.

☒ Life sciences ☐ Behavioural & social sciences ☐ Ecological, evolutionary & environmental sciences

For a reference copy of the document with all sections, see nature.com/documents/nr-reporting-summary-flat.pdf

Life sciences study design

All studies must disclose on these points even when the disclosure is negative.

Sample size	Biochemical data shown were from at least three independent experiments. The sample size was sufficient to represent reproducibility and credibility in the biochemical assay.
Data exclusions	No data was excluded from analysis.
Replication	All experimental findings were reliably reproduced. The data were analyzed from three independent experiments.
Randomization	Randomization was not relevant to biochemical assay and was not performed for all experiments.
Blinding	Blinding was not essential to the biochemical assay.

Reporting for specific materials, systems and methods

We require information from authors about some types of materials, experimental systems and methods used in many studies. Here, indicate whether each material, system or method listed is relevant to your study. If you are not sure if a list item applies to your research, read the appropriate section before selecting a response.

Materials & experimental systems

n/a	Involved in the study	
☒	☐	Antibodies
☒	☐	Eukaryotic cell lines
☒	☐	Palaeontology and archaeology
☒	☐	Animals and other organisms
☒	☐	Human research participants
☒	☐	Clinical data
☒	☐	Dual use research of concern

Methods

n/a	Involved in the study	
☒	☐	ChIP-seq
☒	☐	Flow cytometry
☒	☐	MRI-based neuroimaging
